# CO-CREATING: THE VALUE OF INTERGENERATIONAL ART-BASED EXPERIENCES

**DOI:** 10.1093/geroni/igae098.2940

**Published:** 2024-12-31

**Authors:** Joann Montepare, Deborah Baldizar

**Affiliations:** Lasell University, Newton, Massachusetts, United States; Lasell University, Seekonk, Massachusetts, United States

## Abstract

Intergenerational classroom activities are common in the traditional aging curriculum. However, contemporary higher education initiatives (Age-Friendly University/AFU, Age Inclusivity Domains of Higher Education/AIDHE) advocate for the value of reciprocal intergenerational exchange across the curriculum to enhance the teaching and learning experience. Moreover, as ageism persists there is a need to extend intergenerational contact (a tool for disrupting ageism) for the benefit of all students. This poster will present an art-based classroom case study to show how incorporating intergenerational exchange in classes without a specific aging focus is not only possible, but also an effective educational strategy. In this case, younger students and older adults (residing in a local university-based retirement community) co-enrolled in a Ceramics class over the course of several semesters. Following the classes, a group of younger and older learners were invited to complete a survey about their experience. The survey included open-ended response questions (e.g., about benefits, challenges, how to enhance future classes, etc.) and scaled-response questions (e.g., about how the experience impact age attitudes, the value of intergenerational classes, interest in other intergenerational classes). Younger students’ responses were very positive, reinforcing the positive educational and social values of intergenerational interaction and indicating they would welcome more such class opportunities. Older adults’ responses were likewise positive, noting how the experience nurtured mutual respect and the sharing of different perspectives. Both groups offered useful recommendations for future classes, to be described.

